# Solutions to Big Data Privacy and Security Challenges Associated With COVID-19 Surveillance Systems

**DOI:** 10.3389/fdata.2021.645204

**Published:** 2021-12-15

**Authors:** Vibhushinie Bentotahewa, Chaminda Hewage, Jason Williams

**Affiliations:** School of Technologies, Cardiff Metropolitan University, Cardiff, United Kingdom

**Keywords:** GDPR, Big Data, privacy, surveillance, COVID-19, contact tracing, data protection

## Abstract

The growing dependency on digital technologies is becoming a way of life, and at the same time, the collection of data using them for surveillance operations has raised concerns. Notably, some countries use digital surveillance technologies for tracking and monitoring individuals and populations to prevent the transmission of the new coronavirus. The technology has the capacity to contribute towards tackling the pandemic effectively, but the success also comes at the expense of privacy rights. The crucial point to make is regardless of who uses and which mechanism, in one way another will infringe personal privacy. Therefore, when considering the use of technologies to combat the pandemic, the focus should also be on the impact of facial recognition cameras, police surveillance drones, and other digital surveillance devices on the privacy rights of those under surveillance. The GDPR was established to ensure that information could be shared without causing any infringement on personal data and businesses; therefore, in generating Big Data, it is important to ensure that the information is securely collected, processed, transmitted, stored, and accessed in accordance with established rules. This paper focuses on Big Data challenges associated with surveillance methods used within the COVID-19 parameters. The aim of this research is to propose practical solutions to Big Data challenges associated with COVID-19 pandemic surveillance approaches. To that end, the researcher will identify the surveillance measures being used by countries in different regions, the sensitivity of generated data, and the issues associated with the collection of large volumes of data and finally propose feasible solutions to protect the privacy rights of the people, during the post-COVID-19 era.

## Introduction

The urgent need to manage and find solutions to overcome the effects of the coronavirus necessitates collecting data in large volumes. On the one hand, Big Data acquisition and storage apparently poses a significant threat to the privacy of individuals, and on the plus side, it helps make informative decisions that are crucial for the prevention of COVID-19. Data protection law faces many challenges in the digital age, and the emergence of Big Data is the most conspicuous and challenging. In the Big Data era, the public enjoys many benefits that Internet technology offers to them, but they also do face potential privacy breaches. The failure to protect user accounts and personal data will directly threaten their privacy and security.

The keynote of this paper seeks to support the notion that a pandemic should not be used as a panacea for the introduction of new general surveillance measures without consent. The response of the government and the technology industries to the coronavirus outbreak became headline news, and concerns were raised about the contact tracing apps, mobile location data tracking, and police surveillance drones (Matthan, 2020, 100). Also, new privacy issues have emerged as the organisations started levelling up surveillance using thermal cameras and face-recognition technology in preparation for the resumption of normal working patterns. The governments also have to comply with the use of surveillance tools in combating the pandemic and sought to strike a balance without compromising data privacy laws.

Civil rights organisations, data protection authorities, and research scholars also have highlighted the risk of increased digital surveillance after the pandemic (Gasser et al., 2020, E425–E434). These groups have emphasised the need for having baseline conditions such as lawfulness, necessity, and proportionality in data processing and the need for social justice and fairness (Gasser et al., 2020, E425), and these conditions should be considered before implementing digital surveillance technology. However, the UN holds the view that the use of AI and Big Data for tackling COVID-19 could threaten human rights globally and has expressed concerns about the deployment of data surveillance techniques during the current crisis. It has also underscored the risk in the adaptation of technology in the future becoming the justified norm (Whitehead, 2020).

The data protection regulations of the European Union are based on the premise that the types of data considered to be sensitive require stricter protection than other types due to the higher security risk factor involved in processing them (Kuskonmaz and Guild, 2020). The European Court of Human Rights (ECtHR) has upheld the view that health data must be subjected to stricter safeguards than nonsensitive data (Kuskonmaz and Guild, 2020). The processing of a special category of data is prohibited unless it is carried out for purposes specified under certain conditions (Kuskonmaz and Guild, 2020). The information that is necessary to fight the virus must be up-to-date, should not be retained for longer than required, and should be deleted after the crisis is over without delay (Accessnow, 2020, 14).

The lessons learned in responding to health sector crises in the past show that the deployment of invasive surveillance could be misguided and may have potentially harmful consequences for human rights and public health (Accessnow, 2020, 14). The Big Data tracking systems used during the Ebola outbreak led to violation of privacy rights of millions of people and had minimal effect on the intended purpose to combat the virus (Accessnow, 2020, 14). The presumption here was that the urgency to tackle the outbreak overshadowed the importance of safeguarding the privacy rights of the citizens. Therefore, even during a health crisis, the right balance should be struck to protect the privacy rights of the citizens.

## Deployed Surveillance Measures

The history of surveillance measures goes back to the 14th century plague outbreak in Europe (Tognotti, 2013). Isolation of affected groups and movement restrictions on the population were imposed as constraining measures to control and prevent spreading the plague, and surveillance measures have been used on similar occasions (Tognotti, 2013). During the severe acute respiratory syndrome in 2003, Hong Kong identified clusters of diseases using electronic data systems (Leung et al., 2004). During the Ebola outbreaks in West Africa in 2014–2016, mobile phone data were used to model travel patterns (Wesolowski et al., 2014), and hand-held sequencing devices enabled more effective contact tracing and better understanding of the dynamics of the outbreaks (Quick et al., 2016). Similarly, digital technologies are in use during the COVID-19 pandemic.

The types of tools deployed during the current pandemic are specifically for the purpose of mitigating the risk and preventing the pandemic from spreading to a wider community. The purpose-built tracing tools are in use for measuring spatial proximity between users and tracking their interaction (Gasser et al., 2020, E426). Two closely located smartphones used for proximity tracking help determine whether an infected person and an uninfected person being in closed proximity contributed to the transmission of the virus from one to the other, in which case, the health authorities can take necessary measures to deal with anyone identified positive of the virus (Jalabneh et al., 2020). Proximity tracking technology when used with a smartphone app can be an effective way to reduce the rate of transmission. Also, a large population in developed countries as well as in low and middle-income countries will benefit from installing the app in their smartphones (LMICs) (Hussein et al., 2020a. Trust Concerns in Health Apps collecting Personally Identifiable Information during COVID-19-like Zoonosis). For example, the Singaporean application Trace Together uses Bluetooth connections to log other telephones nearby and alerts those who have been close to an individual diagnosed COVID-19 positive (Gasser et al., 2020, E426). Symptom checkers are tools of syndromic surveillance that collect, analyse, interpret, and disseminate health-related data (Berry, 2018). The Spanish government used this technology that worked in collaboration with the citizens, health professionals, and the private sector to monitor the disease, respond quickly, allocate resources, and minimise or control the outbreaks (Gasser et al., 2020, E426). Quarantine compliance tools enable real-time monitoring to determine whether individuals are symptomatic or nonsymptomatic and are complying with quarantine restrictions (Gasser et al., 2020, E426). One such example is the use of Taiwan’s Electronic Fence application installed mobile phones to track overseas quarantined arrivals (Gasser et al., 2020, E426).

However, the launching of such automated contact tracing applications carries inevitable privacy and security challenges (Hussein et al., 2020b. Digital Surveillance Systems for Tracing COVID-19: Privacy and Security Challenges with Recommendations). It has been reported that the apps could be repurposed to target their users, and jamming, storage, and power drain attacks and active and passive eavesdroppers are such security challenges (Hussein et al., 2020a. Digital Surveillance Systems for Tracing COVID-19: Privacy and Security Challenges with Recommendations). An adversary, on the other hand, can tag an individual’s mobile phone with the contract tracing app to a carrier, which will broadcast false proximity data to the masses. Such actions will lead to wastage of expensive diagnosis resources and are likely to affect the trust in and the efficiency of government mechanisms (Hussein et al., 2020b. Digital Surveillance Systems for Tracing COVID-19: Privacy and Security Challenges with Recommendations).

The world community seems to have made a concerted effort to ease coronavirus lockdown restrictions and create a conducive environment for people to return to normal work patterns. In anticipation of less restrictive measures, employers have started setting up tech measures in the workplace to protect their employees from COVID-19 and avoid related incidents. These measures mean the installation of new surveillance systems including tracking software to identify individuals who may have been exposed to the virus, carry out cleansing, monitor social distancing, and locate them using Bluetooth beacons embedded in their security passes (Chesler, 2020). In addition, cameras with body temperature measuring capabilities are in use to identify infected individuals entering the building (Chesler, 2020). The cameras take a reading close to a person’s eyes; if fever is detected, a warning alert is emitted; then the person can be sent home (Chesler, 2020). This technology is being used by Amazon to check employees at the entrances to its European and United States warehouses as well as to the food store chain (BussinessFirst, 2020).

The use of technical safety applications enables companies to identify contaminated locations once a positive case is found and carry out cleansing procedures quickly (Chesler, 2020). This is a time-saving cost-effective way for the organisation. Also, the managers can be alerted in a circumstance where the number of employees in a congregation is found to be excessive and more than allowed at any one time (Chesler, 2020). Japan has been looking into limiting the number of employees in close proximity to avoid too many warning alerts being sent in closely grouped situations (Chesler, 2020).

Also, many countries use drone technology to control the pandemic. However, the reports suggest that the use of drone surveillance would lead to violation of privacy, especially if the data in the form of image or video is downloaded by an intruder (Hussein et al., 2020a. Digital Surveillance Systems for Tracing COVID-19: Privacy and Security Challenges with Recommendations). The images or video clips of an individual obtained without consent from a drone during an upload or extracted from the cloud server could be used in malicious ways against the individual (Hussein et al., 2020b. Digital Surveillance Systems for Tracing COVID-19: Privacy and Security Challenges with Recommendations). Also, photo image formats such as JPEG contain details of the location and the time photo was taken in the image header files, and some argue that the stolen photos would cause additional damage to personal privacy (Hussein et al., 2020a. Digital Surveillance Systems for Tracing COVID-19: Privacy and Security Challenges with Recommendations).

In the next section, the researcher examines the range of technological solutions that the countries have already taken to tackle the pandemic and the steps taken to respond to the sensitive nature of the data generated. The precursor to developing solutions to alleviate long-term privacy concerns is having a good understanding of different mechanisms in place to do so.

## Specific Measures Taken by Countries From a Regional Perspective

### Asian Region

South Korea refrained from imposing nationwide lockdown or travel restrictions despite the risks from coronavirus incidents in the country (Fahim et al., 2020). But instead, the South Korean authorities have been using different covert tech methods to manage the pandemic. The health authorities resorted initially to track public movement tracking the public and follow up tracing of those diagnosed positive with the use of GPS phone tracking, credit card records, video surveillance, and interviews with the patients (Fahim et al., 2020) (Table 1). As a supplementary measure, South Korean authorities have been sending health advisory texts containing details of infected patients and hyperlinks with details of their movements (Servick, 2020). The reaction to this covert monitoring method aroused strong concerns about the potential breaches of medical confidentiality and inevitable stigmatisation of virus-carrying individuals due to the exposure of their identities in the public domain. Also, according to the reports, patient travel history, excluding the names, was published by the in-country authorities to make others aware of the risks of coming to contact with a person diagnosed positive (Fahim et al., 2020). The authorities also use another smartphone app to monitor thousands of people in self-quarantine and report their movements to the government (Fahim et al., 2020).

**TABLE 1:** Summary of measures taken to tackle the pandemic by countries in the Asian region.

	GPS tracking	Credit card records	Video surveillance	Contact tracing using Bluetooth technology	Face recognition cameras	Mobile network monitoring	Drones
South Korea	✔	✔	✔				
Singapore				✔			
Kazakhstani	✔		✔				
Bangladesh			✔				
China	✔		✔		✔		✔
Hong Kong	✔						
India			✔				
Taiwan						✔	

Singapore also assembled technical measures to contain the epidemic by aggressively tracking chains of infection. The apps for mobile phones were developed to help enforce self-quarantine rules and support contact tracing efforts using “Bluetooth technology” (Fahim et al., 2020) (Table 1). According to the reports, the government of Singaporean has published personal information belonging to coronavirus patients as a warning to those who may have been in close proximity (Franceschi-Bicchierai, N.D). Kazakhstani citizens placed in quarantine use a SmartAstana tracking app, and it enables the officials to ensure that those in quarantine remain in isolation (Gussarova, 2020). By contrast, the city of Almaty Ministry of the Interior relies on video surveillance technology called Sergek, produced locally by the telecommunications firm, Korkem Telecom, to detect individuals breaching quarantine rules (Gussarova, 2020). These two are the only known measures of new surveillance technologies the government uses as antipandemic tools. (Table 1).

The use of CCTV footage to identify people wearing masks had become a challenging task to do. Therefore, in Bangladesh, a local company has developed a CCTV camera feed system for surveillance, to successfully identify COVID-19 infected people and/or those identified positive (DhakaTribune, 2020) (Table 1).

China has been using practically every surveillance system at its disposal (Gershgorn, 2020) (Table 1). Face-recognition cameras are located in public places by the authorities to carry out facial recognition searches and also, mobile phones are used for location tracking (Gershgorn, 2020). Surveillance cameras have also been installed inside private dwellings as well as outside people’s front doors, and the inhabitants are placed under mandatory quarantine (Gan, 2020). The Chinese government is engaged in tracking individuals through smartphone apps such as Alipay and WeChat that grades their health and assigns them a classification of green, yellow, or red (Pisa, 2020). The app transmits these data to the police, and it works as an entry pass to certain public places (Gershgorn, 2020). China has not stopped there and has gone as far as exerting pressure on private companies in the country to hand over data and to support the pandemic containment effort (Gershgorn, 2020). In Hong Kong, airport arrivals are supplied with electronic tracking bracelets that must be synced to their home location by way of the mobile phone GPS signal (Saiidi, 2020).

Indian authorities have expanded tracking citizens through digital and analogue means (Gershgorn, 2020) (Table 1). Location data and CCTV footage are used to track citizens in the southern Indian state of Kerala (Gershgorn, 2020). In addition to personal tracking, Indian authorities are also collecting passenger information from airlines and railroad companies. There has been a case in the state of Madhya Pradesh where the authorities had published the personal information of about 5,400 quarantined people on an online public dashboard but according to reports, it was unintentional (Gershgorn, 2020).

Taiwan uses active mobile network monitoring means to enforce home quarantine for new arrivals or at-risk individuals (Accessnow, 2020, 11) (Table 1). Public authorities receive an alert if an individual’s mobile device happened to be active outside their home (Accessnow, 2020, 11). The reports have also noted that to prevent those under home quarantine from circumventing the measures, public authorities call the number twice a day to ensure that those being quarantined have not abandoned their mobile devices and ventured outside (Accessnow, 2020, 11).

### Middle Eastern Region

Saudi Arabia has taken steps to update two mobile and web-based applications, known as the Mawid (“Appointment”) and the Sehhaty (“My Health”), to respond to the COVID-19 pandemic, by way of a symptom checker enabling people suspected of having COVID-19 to directly book appointments at dedicated COVID-19 clinics and countrywide drive-through mass testing locations (Hassounah et al., 2020). The Health Electronic Surveillance Network (HESN) serves as a national platform for communicable disease surveillance, which is mainly used as a reliable data source for all COVID-19 laboratory tests in the Kingdom (Hassounah et al., 2020). Moreover, the Patient Tracing Unit (Taqasi) platform was implemented to enhance contact tracing tasks around the Kingdom using laboratory results generated from the HESN (Hassounah et al., 2020).

The National Health Emergency Operation Centre had launched a smartphone app Tetamman (“Rest Assured”) to provide preventative and clinical guidelines for home isolation (Hassounah et al., 2020). This app is remotely linked to a smart bracelet that can be used by those individuals returning from abroad, as well as those isolated at home (Hassounah et al., 2020). Two smartphone apps implemented by the Saudi Data and Artificial Intelligence Authority (SDAIA) follow the international Google and Apple guidelines on data privacy in contact tracing (Hassounah et al., 2020) (Table 2). The first is the Tawakkalna, a GPS-enabled app for monitoring and restricting movements of individuals during curfew hours, with the capacity to issue exemption permits. The second is the Tabaud (“Distancing”) for transmitting deidentified data for preventing close contact with COVID-19 confirmed cases (Hassounah et al., 2020).

**TABLE 2:** Summary of measures taken to tackle the pandemic by countries in the Middle Eastern region.

	GPS tracking	Credit card records	Video surveillance	Contact tracing using Bluetooth technology	Mobile network monitoring	Drones
Saudi Arabia	✔					
Iran				✔		
Israel					✔	
Qatar	✔			✔		

The cited report suggests that Iran appears to be using smartphones to track citizens in the fight against COVID-19 (Doffman, 2020) (Table 2). Iranian researcher Nariman Gharib has revealed that the citizens are put under pressure to download an app that would, according to the researcher, help diagnose the coronavirus vector (Doffman, 2020). However, further, the report cited that Google has removed the app from the Play Store, but the Iranian Ministry of Health has assured that no privacy lines were being crossed (Doffman, 2020).

The Israeli government’s domestic security agency, the Shin Bet, is using data from telecom providers to track the locations of millions of citizens to find people diagnosed with the coronavirus and alert those with whom the infected person might have interacted (Scheer and Cohen, 2020). This has raised security concerns amongst many civil liberties groups (Altshuler and Hershkowitz, 2020) (Table 2).

Qatar has made it mandatory for every citizen to download the Ehteraz app and keep it installed indefinitely if they intend to leave their home (Gershgorn, 2020) (Table 2). It requires permission to share data, including location, access to all files, and access to call information (Gershgorn, 2020).

### European Region

The project OASIS (United Kingdom) collects data from third-party app providers who collect information on COVID-19 related symptoms and demographic data to assist the NHS in its pandemic response work (Ministry of Defence, 2020) (NHSX, 2020) (Table 3). The United Kingdom government says Project OASIS will strictly comply with data protection legislation when sharing personal data, and the Ministry of Defence Strategic Command’s technology innovation hub, JHub, has been given the remit to oversee the secure transfer of relevant symptom and epidemiology data from the third-party apps to NHSx (Ministry of Defence, Strategic Command, and jHub Defence Innovation, 2020). A specific role of JHub is to remove any identified information, erase incorrect or duplicate data, and check for security issues (NHSX, 2020).

**TABLE 3:** Summary of measures taken to tackle the pandemic by countries in the European region.

	GPS tracking	Credit card records	Video surveillance	Contact tracing using Bluetooth technology	Mobile network monitoring	Drones
United Kingdom				✔		
Turkey				✔		
Italy	✔			✔		
Germany	✔			✔		
Belgium						✔
France			✔	✔		
Poland				✔		
Bulgaria					✔	

The United Kingdom is reportedly in discussions with telecom companies to engage in tracking its citizens’ location data (Gershgorn, 2020). Also, in the meantime, the National Health Service (NHS, N.D) has partnered with Palantir to track the spread of the virus and its impact on the health system (Gershgorn, 2020). The mobile industry continues to supply location data of individuals to local, state, and federal government organisations to enhance movement tracking (Gershgorn, 2020). The quality of data enables accurate detection of people’s movement and whether they comply with the requirement to stay at home or not by hanging around open public places.

The Turkish government tracks the locations of coronavirus patients using their cellular data, automatically sends warning messages to those detected violating quarantine rules, and the cellular companies operating in Turkey are cooperating with the government in its effort to gather essential data (BIA News Desk, 2020). However, as has been reported, people have become worried about downloading (government-provided) surveillance apps and entering requested information on them (Fahim, Kim, and Hendrix 2020) (Table 3).

The surveillance tool supplier Cy4Gate in Italy is setting up surveillance tools to track every citizen and their contacts to multiple governments around the world, including their own (Franceschi-Bicchierai, N.D) (Table 3). People, by downloading the app and enabling it to track their location as a part of the system, will give voluntary consent (Franceschi-Bicchierai, N.D). According to the reports, Cy4Gate will anonymise the data and only the governmental agency will be able to deanonymise it (Franceschi-Bicchierai, N.D). Immuni is an app used in Italy to control the spread of the pandemic. It is an open-source COVID-19 contact tracing app (Guerrini, 2020). After a testing phase in four Italian regions, the app started being active in the whole country (Reuters Staff, 2020). Immuni has been designed and developed whilst taking great care to safeguard user privacy (Presidenza del Consiglio dei Ministri, 2020). To this end, the app does not collect any information such as first name, last name, date of birth, telephone number, email address, the identity of the people you meet, location, or your movements (Presidenza del Consiglio dei Ministri, 2020).

In Germany, privacy laws allow the government to compel a technology company to share an individual’s location data in the interests of national security (Servick, 2020). The “GeoHealth” app in development relies partly on the location of Google account holder’s data anonymised and stored in a central server, and data analytics would compare users’ movements to those of infected people and send color-coded alerts based on how recently they may have acquired the virus (Servick, 2020) (Table 3). One issue associated with GeoHealth is that the collected data will be stored on a central server and the government can get access to the peoples’ information (Servick, 2020). According to the human right organisation, such as Amnesty International, developing apps based on centralised architecture have an impact on peoples’ privacy, and for that reason, these human rights organisations do encourage to develop apps based on the decentralised architecture (Bentotahewa et al., 2020a. Do Privacy Rights Override #COVID19 Surveillance Measures?) (Robinson, 2020).

In Belgium, authorities use drones used to make announcements and to capture surveillance footage (Limam, 2020) (Table 3). Also, telecoms in Belgium provide data to Dalberg Data Insights (private company) and analyse obtained information to detect widespread trends of movement in the country (Gershgorn, 2020).

France has grave concerns about the potential risk of privacy violations. It is not an obligatory requirement to use the French government’s “Stop Covid” App (Gershgorn, 2020) (Table 3). The security cameras on Paris metro-based systems have the capability to identify those wearing masks and those who are not, and reports point out that these systems are not meant for tracking individuals, but for gathering information on compliance by the commuters (Handler and Liu, 2020) (Table 3).

Poland introduced its own app called “Home Quarantine” with which the quarantined (Polish) citizens were required to check in periodically and send pictures of themselves in their homes, within a 20 min time lapse, and those who failed to do so would incur a fine, effective from 19 March (Gershgorn, 2020) (Nicolas, 2020) (Amnesty International, 2020) (Table 3). This is to ensure that the person is complying with quarantine orders. According to the reports, similar apps are deployed in other countries, including one in India capable of geo-tagging selfies (Amnesty International, 2020). Polish authorities have insisted that this information would remain in government custody for 6 years (Nicolas, 2020). However, there seems to be no explanation of the purpose for retaining the images in government servers for 6 years if it were meant to be a temporary measure.

In Bulgaria, the police authorities at their request have been able to obtain information from telephone and internet operators to monitor the conversations between the citizens. That has allowed them to trace the accurate location of the citizen, monitor those under quarantine, and track the websites visited by the target groups (Vou, 2020) (Table 3). There is a question mark on whether there is a necessity for intrusion into private conversation for the purpose of mitigating the risk of transmission of the virus.

### African Region

In Kenya, mSafari app is being rolled out to help contact tracing (Accessnow, 2020, 10) (Otieno, 2020) (Table 4). As reported, the app would be used to track passengers in public service vehicles including buses, taxis, and other transportation services (Accessnow, 2020, 10). The drivers are expected to download this app and register all passengers (Accessnow, 2020, 10). In addition, the government uses electronic surveillance to track individuals subjected to 14-day self-isolation based on their latest travel history, mainly by monitoring their mobile phone usage activities including geolocations (Ombat, 2020). Also, those in government-imposed self-isolation are instructed to leave their mobile phones switched on and to carry the devices with them (Ombat, 2020).

**TABLE 4:** Summary of measures taken to tackle the pandemic by countries in the African region.

	GPS tracking	Credit card records	Video surveillance	Contact tracing using Bluetooth technology	Mobile network monitoring	Drones
Kenya	✔			✔		
Ghana	✔					
South Africa					✔	

GH COVID-19 Tracker App was developed by Ghana. This app has the capacity to trace those coming into contact with infected person/s (Hussein et al., 2020b) (Table 4). In South Africa, telecom service providers have agreed to share customers’ location data with the government, but it is not yet clear whether it is applicable to location data of confirmed cases only or to the entire population whose information is shared with the government (Business Insider South Africa, 2020) (Table 4).

### American Region

Canadian police have access to a government database and records of people who tested positive for coronavirus, and their personal information is held with them (Gershgorn, 2020) (Table 5). To allow direct access to personal information for community health purposes on a priority basis is understandable but to provide direct access to law enforcement agencies in this way does amount to an invasion of privacy unless permission had been granted for justifiable reasons.

**TABLE 5:** Summary of measures taken to tackle the pandemic by countries in the American region.

	GPS tracking	Credit card records	Video surveillance	Contact tracing using Bluetooth technology	Mobile network monitoring	Drones
Canada				✔		
United States	✔					
Colombia	✔					
Mexico						✔

In the United States, researchers are using Facebook data to measure social distancing. Data collected from Facebook users with their location history enabled and are used to develop maps with aggregated, deidentified location data (Servick, 2020; Lapowsky, 2020) (Table 5). This project presents significant privacy and data protection risks as the users have not given consent to use their location history in the fight against coronavirus and to share their data with researchers.

In Colombia, the CoronApp is used to provide information on the virus (Accessnow, 2020, 16) (Table 5). The application requests a large amount of personal information such as data on ethnicity to function, without transparency as to who would be acquiring the data and how it would be used (Accessnow, 2020, 16).

The government of Guatemala has launched an official application named Alerta Guate with the intent to inform people about COVID-19. To download the app, the users are obliged to allow access to location data and phone microphone and to provide an email address or phone number (Juarez, 2020).

The Mexican government uses drones for surveillance operations (Table 5) of public gatherings and to issue warnings to people. Also, other measures like hand sanitiser gel and face masks are distributed along the public roadsides, in popular neighbourhoods, and on public transport (Tonantzin, 2020).

### Oceania Region

New Zealand uses NZ Covid Tracer App-based user interactions to control and manage the COVID-19 pandemic when the user is diagnosed positive of COVID-19 during sharing his/her credential with the application, and the user is not given the option to raise objections to sharing of personal information. However, it has two-factor authentication and data encryption prior to sending (Hussein et al., 2020a. Trust Concerns in Health Apps collecting Personally Identifiable Information during COVID-19-like Zoonosis). Apparently, the process has a high standard of privacy protection but comparatively, it is not as effective as other contact tracing applications (Hussein et al., 2020b. Trust Concerns in Health Apps collecting Personally Identifiable Information during COVID-19-like Zoonosis).

These overall measures (Table 6) may prove effective in helping contain the outbreak, but at the same token, the governments should ensure that these tools are implemented with full transparency and accountability and with a commitment to cease collection or to reserve exceptional use of data once the crisis had been overcome. The data controllers must still have a lawful and fair basis to collect and use personal data. But privacy experts have raised concerns about how governments were using the data, how it was being stored, and the potential for authorities to maintain heightened levels of surveillance after the coronavirus pandemic is over (Kharpal, 2020). During an extraordinary crisis, many governments appear to be prepared to overlook privacy implications in preference to saving lives as a priority for them.

**TABLE 6:** Summary of the approaches adopted by countries and the reasons.

Region	Approaches to surveillance	Most adopted approaches and reasons
Asian Region	Smartphone app, CCTV, Electronic tracking bracelets and mobile network providers.	It appears that most of the countries in the Asian region are relying on smartphone apps to tackle the pandemic, and the need for expert knowledge to use Smartphone-based apps is not a factor due to their popularity in the region. Also, technologically advanced countries like China and India are seemingly resorting to CCTV technology as well.
Middle Eastern Region	Smartphone apps and telecom providers	Easy access to mobile phones makes the smartphone-based apps commonly used technology.
European Region	Smartphone apps, drones, telecom and internet providers	In comparison to other regions, most of the European countries use well-advanced technologies alongside basic technologies such as mobile phone apps.
African Region	Smartphone apps and telecom service providers	The reliance on smartphone apps to tackle the pandemic is common in many countries.
American region	Government databases, Facebook, smartphone apps and drones	Leaving basic technologies such as smartphone applications aside, some countries in America’s region rely on government databases and data collected from Facebook to manage the pandemic.
Ocean region	Smartphone app.	Despite the high reliance on smartphone apps, there is no sufficient literature about the mechanisms taken by countries in this region.

## Sensitivity of Data Generated and Associated Issues in Collecting Large Volume of Data

Governments have an obligation to guarantee the right to health and to prevent, treat, and control epidemics but it is unlawful to use increased surveillance measures unless strict criteria for doing so can be met. The implemented measures must be necessary, proportionate, and time-bound and are implemented with transparency and adequate oversight to comply with any legal obligations. In the wake of the attacks of September 11, 2001 (9/11), the use of apparatus expanded significantly, and the lessons learned from recent history tell us that there is a real danger of surveillance measures becoming permanent fixtures (Amnesty International, 2020).

The governments in several countries have started to use geolocation data gathered from local telecommunications providers and from social media organisations, Google, and Facebook to monitor the movements of groups of people within a selected certain region (Pisa, 2020). However, name, address, and other pieces of identifying information can be generally removed from these types of datasets, but reidentifying individuals has been proven to be considerably easy unless protected by additional privacy protection (Narayanan and Shmatikov, 2019).

Also, in addition to the risk of reidentification and infringement on personal privacy, digital public health technologies also carry an inherent risk of discrimination (Gasser et al., 2020, E428), and such technologies can be used to collect large amounts of data from the entire population. These data can include race, ethnic group, gender, political affiliation, and socioeconomic status and in turn can be used to demographically classify the population (Gasser et al., 2020, E428). Many of these demographics are sensitive and not necessarily related to a person’s health and might lead to stigmatisation of ethnic or socioeconomic groups. Further, information such as racial demographics might lead to a surge in discrimination, as seen by a rise in attacks on people of Southeast Asian descent in the COVID-19 crisis (Gasser et al., 2020, E428).

The GDPR provides an exception clause to processing of personal data by employers and public health authorities, in epidemic circumstances without having to obtain consent from the data subject (CIPESA staff, 2020). However, obtaining consent validates the legal basis for data processing in compliance with the GDPR. Also, the consent must be obtained from the data subject in an unambiguous statement. Does this mean that for example, proximity-based contact tracing application might rely on obtaining consent? Some experts do argue that obtaining consent would be meaningless unless the data subject is given a choice to object to the processing of his data. This suggests that voluntary participation for contact tracing applications might not necessarily rely on consent as the legal basis to process the data. However, mishandling or abusing the data surveillance work will lead to loss of citizens’ trust in data-based initiatives and jeopardise the government’s effort to control the spreading of the coronavirus.

The Health Insurance Portability and Accountability Act (HIPAA) was set up to protect sensitive health information about patients and to prevent them from disclosure without consent or knowledge of the patients (U.S. Department of Health and Human Service, N.D). One specific area to which privacy regulations were applicable was the health care providers who collect and store health information in electronic form (Hussein et al., 2020a. Digital Surveillance Systems for Tracing COVID-19: Privacy and Security Challenges with Recommendations’). However, HIPAA does not stress on the importance of the consent factor and the right to be forgotten as prescribed in the GDPR, and in such instances, stored private data can be misused after the pandemic (Hussein et al., 2020b. Digital Surveillance Systems for Tracing COVID- 19: Privacy and Security Challenges with Recommendations). There is a policy incompatibility between HIPPA and GDPR, which will lead to likely privacy violations in the future.

There are valid questions that need clear answers; on the assumption that the purpose of collection is justified, what is the time scale for data collection and the retention period, and following up from that when will collected data be deleted. These are the questions arising from the collection of a massive amount of personal data of the citizens. Also, another crucial question is what options are available to the public to contest any unethical purposes.

In a matter of months, millions of people in countries around the world have been placed under surveillance. Governments, private enterprises, and researchers monitor the health, behaviour, and movements of the citizens, often without their consent (Fahim et al., 2020). This enormous effort serves as a necessity to enforce quarantine rules and for tracing the spread of the coronavirus. Epidemiologists and government health officials have been deeply involved in designing coronavirus tracking programs (Fahim et al., 2020).

The groups with interests in privacy issues have been far more concerned about the leading roles taken by the intelligence agencies in gathering personal information (it has been the case in Pakistan and Israel) and when tracing is outsourced to private companies (Fahim et al., 2020). For instance, an Israeli company, NSO, which is an infection-tracking software developer, is well known for designing surveillance tools used by authoritarian governments (CIPESA staff, 2020) for spying on dissidents, journalists, and others and for that reason has come under criticism (Franceschi-Bicchierai, N.D).

The collection of data raises privacy concerns about the implications of using technology once the people are back at work and the likely possibility of them coming under more surveillance. The data generated from these technologies can now be used to target these employees, and as such, some privacy advocates already believe that technical solutions already have put people under over surveillance and feel concerned about their job security (Chesler, 2020).

Another concerning issue is the face-recognition technology using advanced surveillance cameras capturing people’s movements without obtaining consent. Monitoring individuals under suspicion of posing a threat to people’s security and national security is understandable, but inevitably the identity of those going about their daily business is also recorded.

The experts have highlighted the potential for heightened surveillance to continue even after the coronavirus outbreak has been brought under control (Kharpal, 2020). The surveillance technologies once introduced have a habit of becoming permanently embedded in the systems (Amnesty International, 2020; Zhong, 2020) and their use becomes the accepted consequence of living in a world threatened by real threats, mainly cyber and conflict. But we have seen recently that a pandemic such as coronavirus can be even more devastating and combating it has stretched resources to the limit, and the introduction and the use of technology system have become the only effective weapon to combat the prevailing COVID-19 pandemic. That has demanded increased deployment of all technology-based systems to mitigate the potential health risks to the communities and manage the virus before it got out of control. The overarching priority is to protect the people and the nation and taking measures to counter such threats will inevitably infringe on civil liberties.

The aftermath of the 9/11 terrorist attack in New York in 2001 was a game-changer for security services all over the world, and the use of advanced technology became the norm for surveillance purposes (Amnesty International, 2020). Soon after the attack, the US promulgated the Patriot Act and it had a direct impact on democratic liberties, such as the right to protection against unwarranted surveillance (Larsen, 2020; Gasser et al., 2020, E429). This is a good example of how the protection of the citizens overrides the privacy of individuals in extenuating circumstances. But, whilst recognizing the need to take prompt action in critical circumstances, the public should be given a clear indication of the purpose of surveillance operations, the proposed types of data to be collected, time scale, and retention period, and the consequence of not doing so would place those individuals engaged in covert operations in danger. However, collected data should remain in the custody of authorised agencies who should be held accountable for any breaches of confidentiality, and as required by law, they should inform those affected by the breaches. These are GDPR specified requirements that many countries have signed up to.

## The Proposed Roadmap Framework

Since the emergence of COVID-19 in Asia in 2020, it evolved into a global pandemic that spread across every continent beyond borders (UNDP, N.D). To meet the mammoth challenges the world community faced, every nation resorted to implementing a variety of technical solutions to arrest and mitigate the catastrophic impact of the pandemic. However, the collection of the mass amount of data using implemented devices contravenes GDPR principles on privacy security, and Big Data generated in this way could potentially undermine the privacy of people in the long term. Therefore, it is strategically important to develop a postpandemic privacy protection “solution framework” and global level mechanisms set up to manage pandemics, like the COVID-19, in the future.

The purpose of the tables below (Tables 8-16) is to illustrate the methodology used to identify the available mechanisms and to identify privacy risks associated with those mechanisms. The objective is to develop appropriate immediate, medium, and long-term solutions to underpin privacy protection and to help management preparedness of a pandemic like COVID-19 in the future. Referring to our observations and investigations made in each section of the tabulated data, the authors provide a summary of different surveillance systems, their objectives, type of data collected, privacy risks, and implications with the aim of developing consistent solutions for the immediate, medium, and long term.

**TABLE 7:** Issues associated with the collection of large volumes of data during COVID-19.

Issue ID	Issue
4.0.1	Geolocation data gathered from local telecommunications providers, social media organisations, Google, and Facebook to monitor movements of groups of people within a selected region (Pisa, 2020) generated Big data, and additional privacy protection measures should be put in place to protect their privacy (Narayanan and Shmatikov, 2019).
4.0.2	Digital public health technologies can be used to collect large amounts of data from the entire population, but it also has an inherent risk of causing discrimination (Gasser, 2020, E428).
4.0.3	The GDPR sets out legal grounds for enabling employers and competent public health authorities to process personal data in epidemic circumstances without the need to obtain consent from the data subject (CIPESA staff, 2020).
4.0.4	The Health Insurance Portability and Accountability Act (HIPAA) was set up to protect sensitive health information about patients, and to prevent them from disclosure without consent or knowledge of the patients (U.S. Department of Health &amp; Human Service, N.D). However, HIPAA does not stress the importance of the consent factor and the right to be forgotten as prescribed in the GDPR (Hussein et al., 2020. Digital Surveillance Systems for Tracing COVID- 19: Privacy and Security Challenges with Recommendations). Collection, processing and sharing of personal data without the consent have been happening for years. The implementation of the GDPR give the data subject the right to request deletion of gathered data and the data controller is obliged to obtain consent from the data subject in collection of data. Both have come to light in the aftermath of pandemic which necessitated mass collection of information.
4.0.5	The questions arising from the collection of a massive amount of personal data of the citizens are specific; on the assumption that the purpose of collection is justified, what is the time scale for data collection and the retention period, following up from that when will collected data be deleted, and what options are available to the public to contest any unethical purposes.
4.0.6	The collection of data using technology raises privacy concerns and of the implications on people becoming under increased surveillance.
4.0.7	Face recognition technology used in advanced surveillance cameras to track people movements without obtaining consent raises privacy and security concerning issues.
4.0.8	There is a real danger of surveillance measures becoming permanent fixtures (Amnesty International, 2020).

**TABLE 8:** Community surveillance: Immediate and long-term solutions proposed by the researcher.

Surveillance systems	Community surveillance
Mechanisms used	Contact tracing
Mechanism objective/purpose	• Break the pandemic transmission chain. Contact tracing identifies and tracks individuals suspected positive of COVID-19.This allows quarantining individuals in the high-risk category and prone to infection/or ill, to prevent transmission to others(WHO, 2020a. Contact tracing in the context of COVID-19- Interim guidance).
Data types collected	• Name, contact number, locations, and movement of the person (ICO, N.D. Maintaining records of staff, customers, and visitors for contact tracing purposes) (Jalabneh et al., 2020).
Privacy risks and implications	• Difficult or impossible to anonymize user movements and association (Zang and Bolot, 2011).• It is not deemed necessary to collect location data for effective contact tracing (European Data Protection Board, 2020).• Issues associated with architecture. Centralised architecture could compromise all user data (Bentotahewa, Hewage and Williams 2020. Do Privacy Rights Override #COVID19 Surveillance Measures?) (Robinson, 2020).• Authoritarian entities such government, employer or university may have exceptional control over the individual (Howell and Talbert, 2020).• Not all the countries have specified the storage policy (Jalabneh et al., 2020).
Preventive mechanisms applied	• Apple-Google’s joint solution uses Bluetooth technology (Howell, and Talbert, 2020).• Bluetooth signal strength between two user devices tracks the distance between them whether they have been in close contact (Howell and Talbert, 2020).• Track potential contact between users without having to track their locations (Howell and Talbert, 2020).• Reliance on decentralised architecture for storing data collected from user devices (Criddle and Leo, 2020) (European Commission, 2020).
The researcher proposed/immediate solutions	• Transparency (ICO. N.D. Right to be informed) is crucial. Governments should make the users aware of the methods of collecting, processing, and storing data.• Data minimisation is a key principle in GDPR (ICO. N.D. Principle (c): Data minimisation): Users should be made aware of the type of data, and collection restrictions, only what is required.• Help build trust and reduce the risk of an entity contravening privacy regulation.• Countries using a centralised version should be aware of the backlash.• The apps should be used on a voluntary basis, not be compulsory (European Data Protection Board, 2020).• Proper learning should be arranged to overcome any potential cyber-attacks (Hussein et al., 2020. Digital Surveillance Systems for Tracing COVID-19: Privacy and Security Challenges with Recommendations).• App manual should cover the topic of how to be secured from hackers (Hussein et al., 2020. Digital Surveillance Systems for Tracing COVID-19: Privacy and Security Challenges with Recommendations).
The researcher proposed long term solutions	• Crucially important to develop a national-level privacy mechanism guaranteeing the protection of privacy of users especially in a pandemic situation (Hussein et al., 2020. Digital Surveillance Systems for Tracing COVID-19: Privacy and Security Challenges with Recommendations).• Should anonymise data to retain data longer than necessary (ICO. N.D. Principle (e): Storage limitation). If difficult to do so, consent must be obtained from users.• Important to have an international level agreement on the retention period for information collected during the pandemic.• National level decisions will not suffice as pandemic has gone beyond national borders.

**TABLE 9:** At the primary care level surveillance: Immediate and long-term solutions proposed by the researcher.

Surveillance systems	At primary care level surveillance
Mechanisms used	Community testing facilities: (WHO, 2020b. Surveillance strategies for COVID-19 human infection-Interim guidance, 3). • Drive-through sites. • Fixed sites in community buildings.
Mechanism objective/purpose	• To detect individual cases and clusters in the community. (WHO, 2020a. Surveillance strategies for COVID-19 human infection-Interim guidance, 3).
Data types collected	• Generic data: age, sex, location of residence, illness detected date, samples taken and test result (WHO, 2020b. Surveillance strategies for COVID-19 human infection-Interim guidance, 3).Additional information collected by some countries (i.e., UK):• Ethnicity, vehicle registration number, National Insurance number, NHS number, employer details, and of other members of the household (Department of Health and Social care. 2020).
Privacy risks and implications	• In the testing process, a large amount of data is collected (by different countries) and exposed to undue risks of breaches (BBC. 2020) by the hackers thereby allowing them easy access to personal data records.• The samples analysed and results supplied to NPEx by the laboratories are forwarding to the NHS. Given the length of the process chain, chances of human error in transmitting test results in this way potentially impact the individuals (NPEx, N.D).
Preventive mechanisms applied	
The researcher proposed immediate solutions	To keep in line with GDPR guidelines. • Important to collect a minimum amount of information (ICO. N.D. Principle (c): Data minimisation). A better option is to collect optimum data needed at the symptom diagnosis stage, including any other symptomatic health conditions, and voluntary self-declaration of other information such as the vehicle number, ethnicity, and other useful information.• Crucially important to provide training to those who assist in sending final test results (General Medical Council, N.D).
The researcher proposed long term solutions	• Need a global level mechanism/policy in place setting out the maximum allowable time duration for store collected data during the pandemic.• If required to retain data for research purposes, only anonymised data should be used (ICO. N.D. Principle (e): Storage limitation).

**TABLE 10:** Hospital-based surveillance: Immediate and long-term solutions proposed by the researcher.

Surveillance systems	Hospital-based surveillance
Mechanisms used	• Data records taken and reported daily. (WHO, 2020b. Surveillance strategies for COVID-19 human infection-Interim guidance, 3) (Goethem, et al. 2020).
Mechanism objective/purpose	• To identify the spread of the virus and the affected communities (Goethem, et al. 2020).
Data types collected	• Age, gender, and place of residence (WHO, 2020b. Surveillance strategies for COVID-19 human infection-Interim guidance, 3).• Illness onset date, sample collection date, admission data (WHO, 2020b. Surveillance strategies for COVID-19 human infection-Interim guidance, 3).• Type of laboratory test and laboratory test results (WHO, 2020b. Surveillance strategies for COVID-19 human infection-Interim guidance, 3).• Whether a health care worker or not (WHO, 2020b. Surveillance strategies for COVID-19 human infection-Interim guidance, 3).• Condition of the patient, severe or not, at the time of reporting, post-admission medication ventilation or in intensive care unit (WHO, 2020b. Surveillance strategies for COVID-19 human infection-Interim guidance, 3).• Either the discharge date or cause of death, as applicable (WHO, 2020b. Surveillance strategies for COVID-19 human infection-Interim guidance, 3).
Privacy risks and implications	• Breaches of sensitive health information will reveal clinical information of the patients, their inherent health conditions, and the entire medical records (Beltran-Aroca, 2016).
Preventive mechanisms applied	• Blockchain technology has been suggested for use in the health care sector (Seiferty, 2020).
The researcher proposed immediate solutions	• Limit access to the patient medical record.• Back up medical records at least twice a week.• Should not share the identifiable information with the media groups without the patient’s consent (Zhang, 2020).• It seemed important to share information with other organisations, to be aware of the severity of the virus, always choose anonymised data, limited data as much as possible (ICO. N.D. Principle (e): Storage limitation).
The researcher proposed long term solutions	• Need a global level mechanism/policy limiting maximum time duration the authorities are allowed for storing information.• If the authorities are interested in retaining collected data for research purposes, only the anonymised data should be used (ICO. N.D. Principle (e): Storage limitation).

**TABLE 11:** Healthcare-associated surveillance: Immediate and long-term solutions proposed by the researcher.

Surveillance systems	Healthcare-associated surveillance
Mechanisms used	• Take daily figures and report them (WHO, 2020b. Surveillance strategies for COVID-19 human infection-Interim guidance, 3-4).
Mechanism objective/purpose	• To allow rapid control: All cases and clusters in health care settings should be investigated and documented for their source and transmission patterns (WHO, 2020b. Surveillance strategies for COVID-19 human infection-Interim guidance, 4).
Data types collected	• The number of COVID-19 cases and deaths amongst health workers (WHO, 2020b. Surveillance strategies for COVID-19 human infection-Interim guidance, 4).
Privacy risks and implications	• Health information is sensitive information (European Data Protection Supervisor, N.D).• A data breach will reveal medical information, conditions as well as medical history pertaining to any other conditions (Beltran-Aroca, 2016).
Preventive mechanisms applied	
The researcher proposed immediate solutions	• Limit access to the patient medical record.• Back up records at least twice a week.• Should not share identifiable information with the media organisation, or departments in the hospital without the patient’s consent (Zhang, 2020).
The researcher proposed long term solutions	• Important to use only anonymised data if retaining for research purposes, anonymised data should be used (ICO. N.D. Principle (e): Storage limitation).• Need a global level mechanism/policy to obtain consent from the patients before deciding to use personal information by the authorities for research purposes.

**TABLE 12:** Laboratory testing data surveillance: Immediate and long-term solutions proposed by the researcher.

Surveillance systems	Laboratory testing data surveillance
Mechanisms used	• Take daily figures and report them (WHO, 2020b. Surveillance strategies for COVID-19 human infection-Interim guidance, 4).
Mechanism objective/purpose	• To identify the total number of individuals tested for SARS-CoV-2 virus. (WHO, 2020b. Surveillance strategies for COVID-19 human infection-Interim guidance, 4).• To monitor the trends (WHO, 2020b. Surveillance strategies for COVID-19 human infection-Interim guidance, 4).
Data types collected	• The number of tests conducted, and the cases confirmed by each diagnostic method used should be logged and reported (WHO, 2020b. Surveillance strategies for COVID-19 human infection-Interim guidance, 4).
Privacy risks and implications	• Should not reveal identifiable information of COVID-19 positive patients without consent (Zhang, 2020).
Preventive mechanisms applied	
The researcher proposed immediate solutions	• Anonymise the identity of the patient.• Access control.
The researcher proposed Long term solutions	• Need a global level mechanism/policy setting out a maximum time duration allowed for storing of information.• Important to use only anonymised data if retaining for research purposes (ICO. N.D. Principle (e): Storage limitation).

**TABLE 13:** Mortality Surveillance: Immediate and long-term solutions proposed by the researcher.

Surveillance systems	Mortality surveillance
Mechanisms used	• Take daily figures and report them. (WHO, 2020b. Surveillance strategies for COVID-19 human infection-Interim guidance, 4).
Mechanism objective/purpose	• To identify the death rates (WHO, 2020b. Surveillance strategies for COVID-19 human infection-Interim guidance, 4).
Data types collected	• The number of COVID-19 deaths occurring in the community, including in long-term-care facilities. Details collected are age, sex, and location of death (WHO, 2020b. Surveillance strategies for COVID-19 human infection-Interim guidance).
Privacy risks and implications	• The GDPR only applies to information that relates to an identifiable living individual (ICO. N.D. What is personal data?).
Preventive mechanisms applied	• The GDPR only applies to information that relates to an identifiable living individual (ICO. N.D. What is personal data?).
The researcher proposed immediate solutions	• The GDPR only applies to information that relates to an identifiable living individual (ICO. N.D. What is personal data?).
The researcher proposed long term solutions	• The GDPR only applies to information that relates to an identifiable living individual (ICO. N.D. What is personal data?).

**TABLE 14:** Participatory surveillance: Immediate and long-term solutions proposed by the researcher.

Surveillance systems	Participatory surveillance
Mechanisms used	• Voluntary reporting (WHO, 2020b. Surveillance strategies for COVID-19 human infection-Interim guidance, 4-5).
Mechanism objective/purpose	• For self-reporting signs/symptoms to the government, medical staff to remain informed of the extent of the spread (WHO, 2020b. Surveillance strategies for COVID-19 human infection-Interim guidance, 4-5).
Data types collected	
Privacy Risks & Implications	• Even people are coming forward, their privacy should not be compromised.
Preventive mechanisms applied	
The researcher proposed immediate solutions	• It is ideal to design questionnaire/voluntary reporting portals to collect data anonymously.
The researcher proposed long term solutions	• The data which are not important can be deleted after the analysis process.• Important to anonymise data if retaining for research purposes (ICO. N.D. Principle (e): Storage limitation).

**TABLE 15:** Event-based surveillance: Immediate and long-term solutions proposed by the researcher.

Surveillance systems	Event-based surveillance
Mechanisms used	• Formal and informal channels such as online content, radio broadcasts & print media. WHO-led Epidemic Intelligence from Open Sources (EIOS) uses to filter data (WHO, 2020b. Surveillance strategies for COVID-19 human infection-Interim guidance, 4).
Mechanism objective/purpose	• To detect any changes in the overall COVID-19 situation (WHO, 2020b. Surveillance strategies for COVID-19 human infection-Interim guidance, 4).
Data types collected	
Privacy risks and implications	• Revealing the identity and the movement of people would cause critical mental distress to the patients (Bentotahewa, Hewage and Williams. 2020. ‘Security and privacy issues associated with Coronavirus diagnosis and prognosis.’).
Preventive mechanisms applied	• Anonymise the identity.
The researcher proposed immediate solutions	• Sensible reporting by not highlighting any group of people based on their gender orientation, ethnicity, or any sensitive nature (Bentotahewa, Hewage and Williams. 2020. ‘Security and privacy issues associated with Coronavirus diagnosis and prognosis.’).
The researcher proposed long term solutions	• There should be a global level media ethics policy in related to pandemic situations.

**TABLE 16:** Closed settings: Immediate and long-term solutions proposed by the researcher.

Surveillance systems	Closed settings
Mechanisms used	• Daily screening (i.e., daily temperature monitoring) for signs and symptoms for COVID-19 (WHO, 2020b. Surveillance strategies for COVID-19 human infection-Interim guidance, 3-4).
Mechanism objective/purpose	• To identify the carriers of the virus before it spreads amongst the extended communities (WHO, 2020b. Surveillance strategies for COVID-19 human infection-Interim guidance, 3).
Data types collected	• Body temperature of the person (WHO, 2020b. Surveillance strategies for COVID-19 human infection-Interim guidance, 3-4).
Privacy risks and implications	• Some organizations can use or disclose sensitive information, such as health data or temperature monitoring results, to prevent or manage COVID-19.
Preventive mechanisms applied	• Use of automated thermal cameras (Practical Law Data Privacy Advisor, 2020).• Organisations in some countries do not record the temperature readings (Tuttle and McKenzie. 2020).• In some countries, organisations do record temperature readings but not personal information. (Practical Law Data Privacy Advisor, 2020).
The researcher proposed immediate solutions	• Every workplace should have a guidance document to ensure personal privacy when recording temperature readings.• Depending on the country if a workplace is disclosing any sensitive information, the information should be anonymised.
The researcher proposed long term solutions	• Need a global level mechanism/policy in place stating the maximum time duration the authorities can store information.• If the authorities would like to keep data for research purposes it is important to use only the anonymised data (ICO. N.D. Principle (e): Storage limitation).

## Discussion and Author Recommendations

### Need to Revisit Developed Guidance Documents for Global Surveillance During an Influenza Pandemic

In May 2020, WHO released an interim guidance document addressing the risks elements associated with the use of digital proximity tracking technologies that were enacted by the nations in response to COVID-19 (WHO, 2020b). A most notable inclusion is the need to undo extraordinary surveillance activities after a crisis has passed, and for that reason, the WHO should urge governments to enact sunset clauses that would automatically deactivate emergency surveillance measures within a set timeline, unless further legislative action is deemed necessary in response to a specific event.

Global research collaboration for infectious disease preparedness (GLoPID-R) on the other hand has published a protocol on data sharing, and under key principles of data sharing, it noted the importance of ethical requirement and transparency (GLoPID-R, 2017). It is also important to consider the following points when GloPID-R and WHO are revisiting their guidance document on global surveillance during an influenza pandemic.

#### The Proposed Techniques

• Roles and responsibilities of WHO and member states, based on national and regional level data protection mechanisms.
• Reference to new techniques used by different nations and the tendency of implications that would occur if the mechanisms do not align with data protection and privacy.
• Compulsory reporting on privacy and security risk assessment before releasing a new technique.
• Obligatory policy requirement on disclosure of collected information to media, health authorities, or any other party.
• Specification on time limits for holding collected data by member states. This step is crucial in addressing the issue 4.0.5 (Table 7). This will give individuals the confidence they need for sharing personal information with the relevant authorities and help the government approach tackling the pandemic.
• Recommendations to media and healthcare professionals when releasing identifiable information on a case-by-case basis reporting.
• Encourage countries to provide a report on the effectiveness of the deployed devices in instances where the number of reported cases is low and to assess whether there would be added value in future deployment of technical devices to tackle pandemic situations.
• A national focal point for the purpose of reporting privacy violations to WHO will help to address the issue 4.0.3 (Table 7). It is also important to establish a department under the WHO umbrella to monitor the use of technical measures and behavioural effect on individual privacy, track any privacy violation and potential threats to individual privacy, and take urgent impartial actions to prevent escalation of the situations.

#### Lessons Learned

• Outline the roles and obligations of WHO and the Member States in connection with the protection of privacy when using surveillance during a pandemic.
• Clarity on the type of data being collected at different stages of the pandemic. Quantity of data collected should be to a minimum level, where possible. This is will also contribute to addressing the issue 4.0.3 (Table 7).
• A global level mechanism in line with GDPR requirements, to enforce deletion of data within a set time scale; also, a global level agreement that makes the government obliged to justify the purpose for retaining personal data collected during the pandemic, which organisations would be entitled to access information, and what actions could be taken to prevent disclosure of personal identities of individuals.
• There should be a binding international legal agreement with collective involvement of all nations across the globe, with a pledge to develop a global level ethics report that applies to healthcare professionals, media, and any other organisation participating in data handling processes, collection, and storage of personal information of individuals.
• Clear guidance made available on timing to deescalate or cease surveillance activities will help to address the issue of 4.0.6 and 4.0.8 (Table 7).
• A defined limit to what extent technology can intervene in people’s privacy will help to address the issue 4.0.6 (Table 7).
• The norms and obligations on violations of personal privacy must be underwritten by legal procedures.

### Proposed Feasible Solutions to Overcome Potential Security Concerns to Protect Citizens’ Right to Privacy During Post-COVID-19

An influenza pandemic will affect every country. Therefore, it is essential to have a standardised coordinated information-sharing mechanism at the global and national levels to effectively manage a serious incident (such as COVID-19). At the national level, authorities need to have informed knowledge of the momentum of the pandemic and awareness of the potential risks not only in their own country but also in the neighbouring countries and in the regions. That approach requires having in place appropriate surveillance systems for gathering personal data, and to avoid contravening laws in member states, there should be laws on surveillance, data collection, storage, and reporting, and confidentiality of the patients must be followed. The patients must be informed of the reasons for sampling and processing of the specimens, and they must be made aware of the benefits that would bring with recommended good practice; also, they should be reassured of their safety and confidentiality in the process itself.

The question is whether there is a strong case for inducing technology into surveillance operations in extenuating circumstances where public safety and security matters, regardless of the nature of the risks. The answer must be a clear “Yes” and can be justified because surveillance plays an important role in times of a pandemic situation or incidents threatening the security of the state. Having all that said, in the context of the COVID-19 pandemic, the enforcement of new digital surveillance powers can also threaten privacy, freedom of expression, and freedom of association and can also lead to skepticism and loss of public trust in the system itself, further undermining the effectiveness of intended public health response. To avoid such misconceptions, it is essential to clearly set before the public the purpose for the imposition of such measure and why they are needed, and arbitrary measures are contested by the activists, as has been seen during the recent health crisis.

There should also be a plan to address post-COVID-19 privacy implication issues associated with the technical solutions and how the public can be reassured of their confidentiality in the long term and the pandemic is not used as an excuse to retain surveillance measures indefinitely. That must be embedded in the decision-making process in accordance with the accepted ethical norms to “obtain consent” before retaining and disclosing personal data collected during the pandemic. One option available to countries for data collection is to have strict data privacy laws when requesting telecommunications and other tech companies to share anonymous, aggregated information in their possession.

The United States and the European Union have specific laws to regulate data collection from the app and device users (Servick, 2020). In the first instance, the collection of data is conditional on consent being obtained from the user of the apps and devices by the collecting organisation (collector) (Servick, 2020). However, the requirement for obtaining consent will not apply to face-recognition technologies. Beyond that, the collector specifying the purpose, process, usage, storage, and disclosure procedures, particularly sharing of personal data, will at least address the issue of 4.0.7 up to some extent. The mobile carriers in Germany and Italy have started to share cell phone location data with health officials in an aggregated, anonymised format (Servick, 2020) (Table 7). Even though individual users are not identified, the data could reveal their general trends, track and trace their gathering locations, and take action to prevent the infection from spreading. The Ministry of Health of Germany, according to reports, has drafted changes to their Infection Protection Act to enable tracking of people suspected of being in contact with coronavirus infected individuals (Digital Health and Care Institute, 2020).

The introduction and adoption of the contact tracing apps and the use of drones and CCTV were considered essential tools to control the virus, prevent transmission within the communities, and reduce the additional burden on the already overstretched healthcare sector. But the low level of focus on privacy and security implication on the citizens and the lack of foresight on legal aspects masked the importance of surveillance measures. It is right to point out that compliance with legal obligations to protect confidentiality and personal privacy is also important in managing the pandemic, and the responsibility for that rests with the government. Therefore, the emphasis should be to strike a balance to successfully manage the pandemic without infringing individual privacy.

Privacy-by-design can help address the risks. Privacy-by-design seeks to deliver the maximum degree of privacy by ensuring that personal data protection is built into the system, by default. For example, privacy-by-design may involve the use of aggregated, anonymised, or pseudonymous data to provide added privacy protection or deletion of data once its purpose is served. For example, the COVID-19 app developed by the Norwegian Institute of Public Health is designed to store location data for 30 days only (OECD, 2020a. Tracking and tracing COVID: Protecting privacy and data whilst using apps and biometrics). Simultaneously, data minimisation principles specify that organisations should collect required information only (ICO, N.D), and it provides a solution to the issue 4.0.2 (Table 7), for instance, when the identities of the employees suspected of having coronavirus symptoms is needed and to know whether they had been exposed to risk whilst visiting any high-risk country. However, collecting information about their household members as far as the workplace is concerned is contestable. The organisations should exercise caution and ensure appropriate data safety security measures are in place when collecting other health data.

RAND Corporation researchers also have developed a concise, standardised, and transparent privacy scorecard that would help health officials understand and evaluate the privacy implications of mobile surveillance programs (Boudreaux, et al., 2020). Also, the availability of a wide range of mobile surveillance programs capable of monitoring COVID-19 had been their intention to have a standardised approach. The RAND wanted public health agencies to be able to compare the efficacy and usability of such programs as well as the inclusion of privacy protection means in different programs that will help make decisions appropriate to intervention selection (Boudreaux, et al., 2020). For example, Australia’s COVIDSafe contact tracing program fulfilled 16 of the 20 scorecard criteria and partially did two others; but in contrast, South Korea’s contact tracing program fully or partially met only six and failed in nine; the remaining five were either unclear or not applicable (Boudreaux, et al., 2020).

Privacy and security researchers are engaged in producing a package of protection mechanisms that would provide the basis for developing a consistent and meaningful privacy protection policy. For example, Harvard University’s Centre for Ethics, in a recent publication, has identified tracing protocols with the capacity to mitigate privacy risks and promote the use of critical security and privacy controls that would enable acceleration of medical responses whilst maintaining people’s rights (Sharma and Masooda, 2020. 1,166). Another team of researchers has come up with a system that has the ability to secure privacy-preserving proximity tracing at a large scale (Sharma and Masooda, 2020. 1,166). It is aimed to help application of anonymous identifiers and functional requirements of fundamental security and privacy, such as data minimisation and retention (Sharma and Masooda, 2020. 1,166). Also, emerged in other publications are suggestions for anonymisation and encryption to generalize peoples’ data whilst at the same time protecting user privacy (Sharma and Masooda, 2020.1165). However, recent research suggests that, despite the anonymisation of personal data, people could still be identified by a limited set of data points (OECD, 2020b. Ensuring data privacy as we battle COVID-19) (Almeida, et al., 2020).

Also, risking privacy violations will reflect badly on accountability and public trust in the government. The possibility of violating one’s privacy by state officials or technology companies might make citizens reluctant to come forward for COVID-19 testing, downloading public health-oriented mobile phone apps, or sharing symptom or location data. More broadly, actual, or perceived privacy violations might discourage citizens from believing government messaging or complying with government warnings and enforceable regulations concerning COVID-19. Therefore, it is imperative to have a privacy governance mechanism in place if the citizen were to have faith in the government and confidence in the actions being taken to mitigate the risk of the pandemic from spreading.

Also, ignoring privacy will have a negative impact on accountability and public trust in the government. The possibility of violating one’s privacy by state officials or technology companies might make citizens reluctant to come forward for COVID-19 testing, downloading public health-oriented mobile phone apps, or sharing symptom or location data. Therefore, it is imperative to have a privacy governance mechanism in place if the citizen were to have faith in the government and confidence in the actions being taken to mitigate the risk of transmission.

Privacy enforcement authorities (PEAs) also have a leading role to play. By proactively advising governments on proposed new legislation, they can ensure clarity in the application of existing privacy and data protection framework (OECD, 2020a. Ensuring data privacy as we battle COVID-19). PEAs in Argentina, Australia, Canada, Finland, France, Germany, Ireland, New Zealand, Poland, Slovakia, Switzerland, and the United Kingdom have issued general guidance to the data controllers and processors, about the application of privacy and data protection laws during the pandemic (OECD, 2020b. Ensuring data privacy as we battle COVID-19).

As a result, many countries have recently passed or are about to pass laws incorporating guidance specific to data collection restrictions, time limits, and the purpose for the collection. For example, the Italian government published a decree to create a special legal framework for collecting and sharing health-related personal data by the health authorities and their associated partners in the private sector, with set guidelines on time limits during the state of emergency (OECD, 2020a. Ensuring data privacy as we battle COVID-19). The German government has proposed amendments to the Infection Protection Law, thereby allowing the Federal Ministry for Health to request persons at “risk” to self-identify and provide their travel history and contact details (OECD, 2020b. Ensuring data privacy as we battle COVID-19).

The announcement made by the Information Commissioner’s Office (United Kingdom) noted the public interest in the application of its data protection law and the urgent need to enable data controllers to balance their obligations to respond to public requests (OECD, 2020a. Ensuring data privacy as we battle COVID-19). The introduction of additional privacy protection measures will protect people’s privacy during the pandemic and will provide a solution to the issue 4.0.1 (Table 7). However, there are other governments that have collected and processed COVID-19-related geolocation data without the need to adopt new legislation. In the Republic of Korea, for instance, the authorities do have existing extraordinary powers to collect personal data, when it is necessary to prevent infectious diseases and prevent the infection from spreading (OECD, 2020a. Ensuring data privacy as we battle COVID-19). In Singapore, personal data can be collected, used, and disclosed without consent to enable contact tracing and other measures in response to an outbreak such as it has been in the case during the pandemic (OECD, 2020b. Ensuring data privacy as we battle COVID-19).

## Conclusion

There is much interest in privacy as nations are engaged in collecting massive amounts of personal data of their citizens in response to the COVID-19 pandemic. The positive steps taken by individual states or collectively by groups of states, the European Union being one, demonstrate the importance of regulating data collection and the impact on the privacy rights of the citizens. There may be a justification for gathering, storing, processing, and sharing personal data; however, COVID-19 must not be a panacea for collecting personal data in this way. The most concerning is the risk of unauthorised disclosure of personal data for unethical purposes and that makes the case for having unambiguous laws to prevent infringements on the privacy of individuals. The failure to do so will affect the credibility of the Big Data collection process, and the public will become even more sceptical and lose faith. The lack of clarity in the purpose for collecting a large amount of data in the first place and what happens to the collected data once the pandemic is over has become an issue for public scrutiny. Therefore, to allay any concerns and fears in the minds of the public, the onus is on the collectors, governments, and organisations to reassure the public that their personal information will remain confidential and secure from unauthorised access.

There are initiatives that the world as a whole or as individual states can take to institutionalise data protection and privacy laws. That requires consensus amongst the states and a commitment to developing a legal framework containing a set of data protection and privacy principles, purpose limitation and data minimisation, and personal data handling that governments and companies should follow. Personal data access limitation, data security, data retention, and research purposes should be the determining factors for collecting data, and the use of personal data should be conditional on public-interest purposes only. Also, personal health data should not be sold or transferred to third parties unless their involvement specifically serves the public interest. An organisation collecting new categories of personal data from individuals and using such data for new purposes should update privacy notices to reflect the new changes in the collection of data. The data handling organisations should keep under constant review existing privacy notices and ensure that they provide up-to-date information of data collected and the purposes for processing. It is the sharing of covertly gathered personal data by organisations without any reference to the data subject that is the most concerning to the public and should be addressed in earnest.

The reality is that the prevailing coronavirus crisis is here to stay for some time yet, and the concerns about surveillance methods the governments and companies are putting in place will not go away either. It is important to have the right laws to cover a range of issues concerning the data collection process and handling of collected data in the long term. However, given the trends and uncertainties associated with COVID-19, it remains to be seen whether after this pandemic the world will be more tolerant than before to the surveillance approach of governments and other organisations. However, to be prepared for any privacy concerns likely to arise in the long term and to be response ready to face future health crisis similar to that of COVID-19, it is crucially important to develop a global level framework that will be readily available to protect individual privacy during a pandemic.

The national level privacy mechanism should provide safeguards and guarantee the protection of privacy of its citizens. On a wider scale of a global pandemic, it is important to have an international level framework or mechanism that clearly defines time limits for the retention period of information collected during the pandemic and requirements for anonymisation of data when transferring to or sharing with a third party or when retaining for research purpose. It is also important to include healthcare-related norms and media ethics in pandemic situations.

## Data Availability

The raw data supporting the conclusions of this article will be made available by the authors, without undue reservation.
